# Implementation of a Wideband Microwave Filter Design with Dual Electromagnetic Interference (EMI) Mitigation for Modern Wireless Communication Systems with Low Insertion Loss and High Selectivity

**DOI:** 10.3390/mi14111986

**Published:** 2023-10-26

**Authors:** Abdul Basit, Amil Daraz, Guoqiang Zhang

**Affiliations:** 1 College of Information Science and Electronic Engineering, Zhejiang University, Hangzhou 310027, China; abdulbasit@nbt.edu.cn (A.B.); amil.daraz@nbt.edu.cn (A.D.); 2 School of Information Science and Engineering, NingboTech University, Ningbo 315100, China

**Keywords:** wideband filter, wireless communication, dual stopband filter, low insertion loss, uniform transmission line, high selectivity

## Abstract

By leveraging the advantages of the uniform transmission line, this manuscript presents a broadband high-selectivity filter range starting from 2.5 GHz to 16.8 GHz, utilizing a simple uniform transmission line structure loaded with three-quarter-wavelength stubs. The proposed UWB filter is studied using the ABCD network parameter method. After that, a shorted T-shaped stub-loaded resonator is coupled with the transmission line of the UWB filter to obtain dual-notch features at 4.4 GHz (for long distance wireless ISPs (WISPs), 4G/5G operator for LTE backhaul) and 7.5 GHz (for X-band downlink communication). The overall footprint is specified as 22.5 mm × 12 mm or 1.12 λ_g_ × 0.6 λ_g_, where λ_g_ represents the wavelength at the central frequency. The operating principle of such a filter is explained, and its controllable broadband response, as well as controllable stopband frequencies, are optimized to show some of the attractive features of the new scheme, such as a super wideband response of about a 148.18% fractional bandwidth; an out-of-band performance up to 25 GHz; five single-resonator transmission poles filtering behaviour at different frequencies, with highly reduced radiation losses greater than 10 dB; a simple topology; a flat group delay; a low insertion loss of 0.4 dB; and high selectivity. Additionally, the filter is fabricated and evaluated, and the results show a good match for experimental validation purposes.

## 1. Introduction

Bandpass filters are the main elements used in communication systems for signal receiving and processing. Bandpass filters were initially designed using a common operating mechanism, involving cascading HP or LP filters using MMRs with central frequencies equal to quarter or full wavelengths. However, with these techniques, the design topology becomes pricey and complex. The construction of a planar microstrip transmission line may be able to circumvent the aforementioned mechanisms because of its simple design, low cost, and easy fabrication process. Wide pass-band filters are becoming increasingly recommended due to their compatibility with integration into various circuits and antennas, which helps to enhance the performance of radio systems [1,2,3]. Over the past few decades, microwave researchers have adopted a few approaches for designing wideband filters [4,5,6,7,8,9,10,11,12,13,14]. Funnel-type ASIRs and DGSs (defected ground structures) were used to design wideband bandpass filters (BPFs) with upper wide stopband responses [4,5,6,7]. In [8], a filter based on a circular quadruple/quintuple-mode resonator with fractional bandwidths (FBWs) of 60% and 62% was created using parallel-coupled microstrip lines. These topologies, however, showed a high IL. By employing interdigital strip lines and grating arrays to attain a respectable FBW of roughly 177%, a wideband filter with a frequency range of 0.29 GHz to 4.82 GHz was designed by the authors of [9]. While this design offered a wide bandwidth, it had a complex geometry, which may introduce challenges in practical implementation. The authors of [10] use interdigital lines and DGSs to develop and construct a wideband BPF on the back of the Rogers RO-4350 substrate that operates between 2.2 GHz and 7.6 GHz, with improved upper-frequency band suppression. The filter’s upper stopband suppression was greatly improved up to 32 GHz by achieving four transmission zeros (TZs) in its passband. The given filter, however, makes use of an intricate design that can lead to manufacturing measurements being costly. In [11], the authors constructed a filter with a fractional bandwidth of 62.3% using a staircase resonator. In a recent study [12], a new H-type sandwich topology was utilized to achieve a broad bandwidth filter response with a fractional bandwidth (FBW) of approximately 132%. The addition of source-to-load coupling improved the filter’s selectivity, but it also increased the insertion loss (IL) in the passband. The overall footprint of the structure covered an area of around 32 × 15 mm. Another research work [13] implemented a high-selectivity UWB filter based on a tapered transmission line resonator (TTR). The design employed a sophisticated geometry to achieve IL < 1 dB and RL > 17 dB. Despite reducing the circuit size and achieving good control over the bandwidth, there is still potential for improvement in the passband performance of the TTR-based UWB filter. In [14], a filter with a good passband was created by combining a low- and high-pass filter. The topology developed an acceptable return loss and a good insertion loss of 0.4 dB. The FBW, however, was only 107%, which was not particularly high. One of the advantages of this prototype is to block unwanted signals up to 20 GHz. Now, to ensure compliance with FCC UWB range regulations and mitigate interference from other frequency bands, the authors of the references [15,16,17,18,19,20,21] have proposed various methods to achieve UWB filters with stopband characteristics such as SIRs, ASIRs, and DGSs, respectively. It is interesting to note that several studies have also been conducted to realize UWB filters with low insertion loss and stopband characteristics. For example, in [22,23,24], UWB filters with low insertion loss were achieved by cascading low-pass and high-pass filter sections. This approach allows for effective filtering across a wide frequency range. UWB filters based on stub-loaded multimode resonators were implemented in [25,26,27,28,29,30,31,32]. These resonators utilize stubs to introduce additional resonant modes, enabling the design of compact filters with desirable UWB characteristics. The stopband characteristics of UWB filters have been studied using techniques such as step impedance resonators (SIRs) [33,34] and multimode resonators (MMRs) [35]. However, some of the reported designs have larger circuit dimensions, which may pose challenges in practical implementations. To address the circuit dimension problem, UWB notch filters based on defected ground structures (DGSs) and defected microstrip structures (DMSs) were proposed in [36,37,38,39]. However, achieving both sharp selectivity and a wide bandwidth simultaneously proved difficult with these techniques. 

Overall, it remains a challenging task for microwave researchers to design UWB bandpass filters with notch bands that have simple topologies and wideband characteristics while maintaining good selectivity. It is worth noting that the techniques mentioned in the literature provide valuable insights into the development of UWB filters, but researchers continue to explore new approaches to overcome the challenges and achieve the desired filter performance. In this regard, a SWB-BPF (super wideband bandpass filter) with a range of 2.5 GHz to 16.8 GHz and five transmission poles using a simple UTL has been developed in this study. Later, a quarter-wavelength resonator with a T-shaped structure is coupled to the SWB filter to suppress unwanted signals for 4G/5G operators for LTE backhaul and X-band downlink satellite communication applications. The proposed wideband filter topology also covered the basic requirement of the ultra-wideband BPF defined by the Federal Communication Commission (FCC), i.e., 3.1 GHz to 10.6 GHz. The filter footprint is about 22.5 × 12 mm, with an absolute bandwidth of 14.3 GHz. Finally, the presented filter was designed and analysed in a 3D EM software, HFSS-15 (High-Frequency Simulator Software) [40], then fabricated on a low-loss PCB. After optimization, the design verifies its attractive features, such as controllable bandwidths and stopbands, a flat group delay, multiple transmission poles, a good return loss, a good roll-off rate, and a wide passband response. 

## 2. Methodology of the Proposed SWB-BPF 

**Figure 1:**
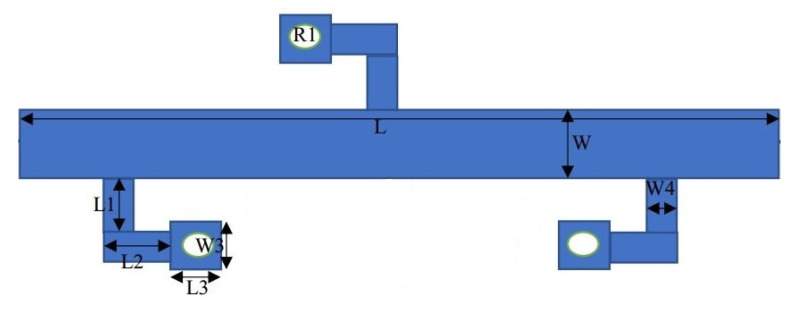
Proposed SWB-BPF architecture.

**Figure 2:**
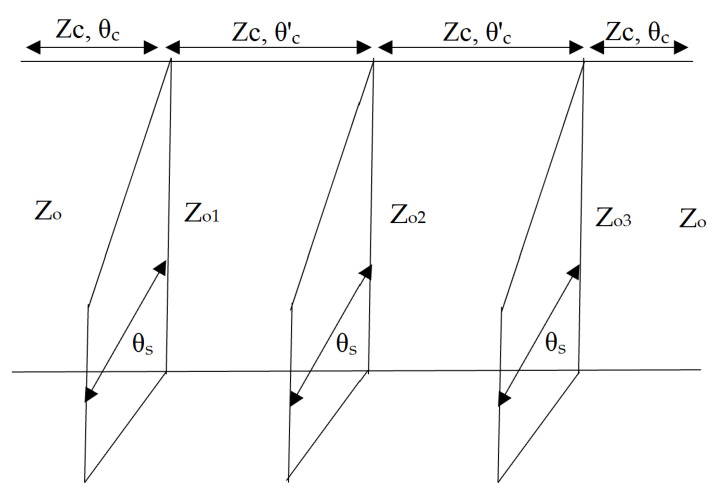
Equivalent configuration of the proposed prototype.

**Figure 3:**
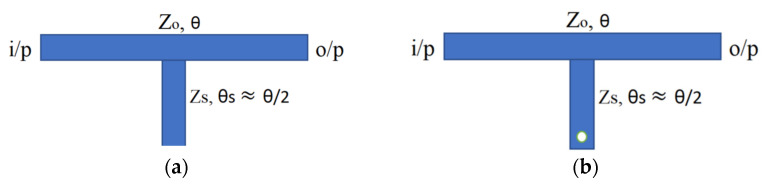
(**a**) Open-stub configuration connected to UTL; (**b**) short-circuited stub connected to UTL.

Figure 1 depicts the SWB-BPF architectural layout. It is made up of a UTL that has three-quarter wavelength stubs loaded onto it; one of these stubs is attached to the upper portion of the UTL, and the other two are positioned symmetrically on the lower portion. The SWB-BPF has been investigated by considering a lossless transmission line, and ignores the effect of inductance and capacitance at short-circuited stubs and at the edges of the junctions, which appears due to the step discontinuities. The equivalent configuration of the initial prototype is shown in Figure 2. *Z*_1_, *Z*_2_, and *Z*_3_ represent the characteristic impedances of the shorted stubs with electrical length of *θ_s_*, separated with connecting lines of electrical lengths of *θ_c_* = 2*θ_s_*. All impedances (*Z*_1_ = *Z*_2_ = *Z*_3_) should be equal for design simplicity. The following steps are used to achieve the extreme broadband response. At first, the resonator with open- or short-circuited stubs, as shown in Figure 3a,b, is connected in the middle of the UTL.

The input admittance (*Y_in_)* can be found by using the ABCD matrix method to obtain the network resonance condition, assuming no losses in the transmission line [41,42].

(1) $$Y_{in}=\frac{1}{Z_{11}}=\frac{C}{A}=0$$

where *Z*_11_ shows the input impedance of the resonator. In Equation (1), when elements *C* and *A* in the transfer matrix have different zeros and *A* does not have poles that are different from those in *C*, this condition ensures the resonance requirement for the resonators in this context.

(2) C=0

when *C* = 0, then *Y_out_* also becomes zero. Therefore,

(3) $$Y_{out}=\frac{1}{Z_{22}}=\frac{C}{D}=0$$

The generalized equation for the left portion of Figure 3a in terms of the matrix elements *A′*, *B′*, *C′*, *D′* is as below [43].

(4) $$\left(\begin{array}{cc} A & B\\ C & D \end{array}\right)=\left(\begin{array}{cc} 1+2B{}^{\prime }C{}^{\prime } & 2A{}^{\prime }B{}^{\prime }\\ 2C{}^{\prime }D{}^{\prime } & 1+2B{}^{\prime }C{}^{\prime } \end{array}\right)$$

The resonance conditions in Equation (4) are same as in Equation (2), and fulfil the following two criteria:

(5) $$C{}^{\prime }=0$$

(6) $$D{}^{\prime }=0$$

The above criteria are easier to analyse as compared to the conventional method, such as *Y_in_* = 0. Now, formulate the resonance equations (*A′B′C′D′*) for Figure 3a,b, which is equal to the input admittance (*Y_s_*) of the stub and the transfer matrix (*abcd*) of the left section of the TL segment [44,45]:

(7) $$\left(\begin{array}{cc} A{}^{\prime } & B{}^{\prime }\\ C{}^{\prime } & D{}^{\prime } \end{array}\right)=\left(\begin{array}{cc} a & b\\ c & d \end{array}\right)\left(\begin{array}{cc} 1 & 0\\ \frac{Y_{s}}{2} & 1 \end{array}\right)=\left(\begin{array}{cc} a+\frac{bY_{s}}{2} & b\\ c+d\frac{Y_{s}}{2} & d \end{array}\right)$$

Here, the matrix (*abcd*) denotes a portion of a non-uniform segment. Using Equation (3), the above equation becomes

d=0

$$Y_{s}=\frac{-2c}{d}$$

When a segment with a uniform TL is used, its *θ* and *Z_o_* become

(8) $$\left(\begin{array}{cc} a & b\\ c & d \end{array}\right)=\left(\begin{array}{cc} \cos\frac{\theta }{2} & jZ_{o}\sin\frac{\theta }{2}\\ Z_{o}{}^{-1}j\sin\frac{\theta }{2} & \cos\frac{\theta }{2} \end{array}\right)$$

By applying resonance conditions, it becomes

(9) $$\cos\frac{\theta }{2}\;\mathrm{where}\;\theta_{n}=\pi ,3\pi ,5\pi$$

(10) $$Y_{s}=-j2Z_{o}{}^{-1}\tan\frac{\theta }{2}$$

Equation (10) determines the resonant electrical lengths (*θ*) for the other component, which relies on the stub parameters. The same analysis can also be used for Figure 3b. In this case, Equation (9) remains unchanged, while Equation (10) becomes

$$-Z_{s}{}^{-1}\cot\theta_{s}=-2Z_{o}{}^{-1}\tan\frac{\theta }{2}$$

**Figure 4:**
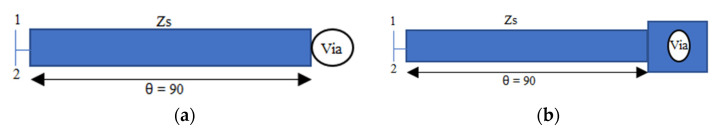
(**a**) Conventional stub. (**b**) Proposed shunt stub.

**Figure 5:**
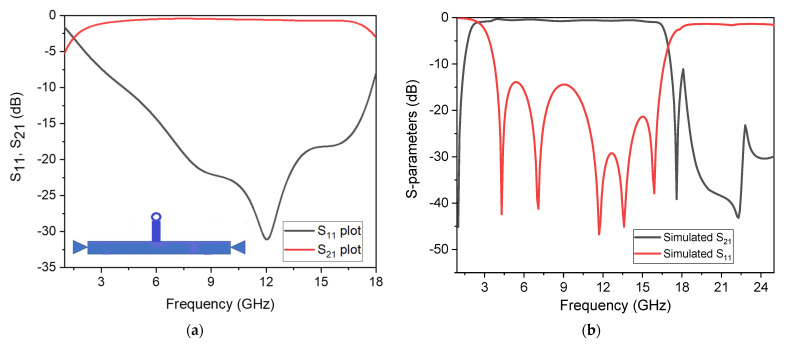
S-parameter responses: (**a**) response of the wideband filter loaded using a single conventional stub; (**b**) response of the proposed broadband filter.

The stubs depicted in Figure 3a,b have been substituted with the stubs illustrated in Figure 4a,b. Initially, a conventional stub, as depicted in Figure 4a, was positioned at the centre of the UTL using matrix Equation (11) [45]. The given configuration generates a wideband response with poor selectivity, with one TP observed at 12 GHz, as depicted in Figure 5a.

(11) $$[A]=\left(\begin{array}{cc} 1 & 0\\ j\frac{1}{Z_{s}}\tan\theta & 1 \end{array}\right)$$

To increase the design flexibility, the stub in Figure 4a is replaced with a stub shown in Figure 4b. Furthermore, two more identical stubs are placed on the opposite side of the transmission line. This adjustment leads to a modified configuration, as depicted in Figure 1. The corresponding equation of Figure 4b is given below.

(12) $$[A]=\left(\begin{array}{cc} 1 & 0\\ -j\frac{1}{Z_{s}}\cot\theta & 1 \end{array}\right)$$

The way the stubs are arranged significantly enhances the filter’s performance, as demonstrated in Figure 5b. The filter exhibits a wide and flat passband, excellent IL, and sharp rejection levels at the lower and upper stopband, with good out of band suppression up to 25 GHz, respectively.

In Figure 1, when the source and load impedances (*Z_o_*) are well matched, then the relation of the reflection coefficient, |*S*_11_|, and power transmission coefficient, |*S*_21_|, can be obtained, and are given below:

(13) $$S_{11}=\frac{A+(B/Z_{o})-{CZ}_{o}-D}{A+(B/Z_{o})+{CZ}_{o}+D}$$

(14) $$S_{21}=\frac{2}{A+(B/Z_{c})+{CZ}_{c}+D}$$

## 3. Mathematical Modelling of the Dual-Notch Filter

**Figure 6:**
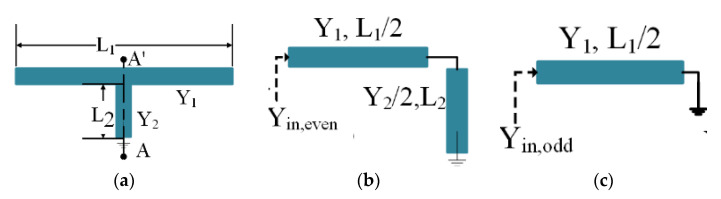
(**a**) Quarter-wavelength T-shaped resonator; (**b**) equivalent even mode; (**c**) equivalent odd-mode model.

In this section, the dual-notch characteristics using a short-circuited, T-shaped, dual-mode resonator are achieved. A symmetrical quarter-wavelength resonator is used instead of a half-wavelength resonator to make the structure compact, as shown in Figure 6. To analyse the behaviour of the structure, the design utilizes a classical method called even–odd mode analysis. This involves separating the circuit into its even and odd modes and analysing each mode separately. This method helps to predict the controllable operating bands of the filter. The even mode will have a symmetric current distribution and can be represented by even terms in the analysis, as shown in Figure 6b, while the odd mode will have an anti-symmetric current distribution and can be represented by odd terms, as shown in Figure 6c, respectively. By applying this analysis and using the basic concept of microwave transmission lines, the equations of the characteristic input admittance of each resonator can be derived, taking into account the lengths of the half-wavelength line (*Y*_1_, *L*_1_) and quarter-wavelength line (*Y*_2_, *L*_2_), with a load admittance (*Y_L_*) [46,47].

(15) $$Y_{in}=Y_{0}\frac{Y_{L}+jY_{0}\tan\theta }{Y_{0}+jY_{L}\tan\theta }$$

Here, *θ = βL*, so the above equation becomes

(16) $$Y_{in}=Y_{0}\frac{Y_{L}+jY_{0}\tan\beta L}{Y_{0}+jY_{L}\tan\beta L}$$

**Figure 7:**
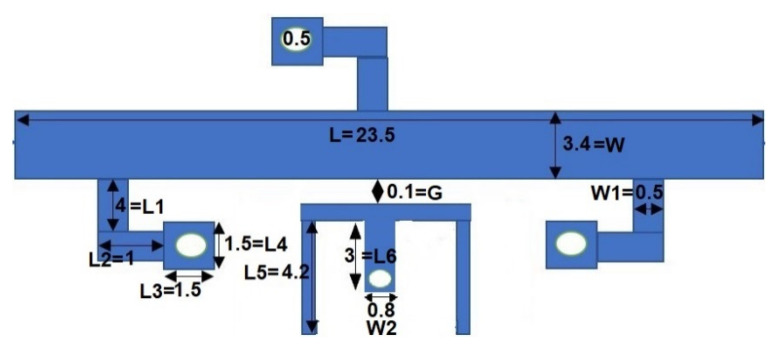
Proposed dual-notch filter.

The next discussion is about how the stopbands are formed. Using the mathematical analysis discussed above, the theoretical resonant frequencies for each stopband are calculated. The first stopband, formed through the fundamental even mode and its resonance frequency, are calculated using the parameters provided in the Equation (19) denominator. Substituting the relevant length values from Figure 7 into Equation (19) and referencing Figure 6b yields a theoretical frequency of 4.18 GHz for the even mode, while the simulation shows 4.4 GHz. The second stopband is generated through the fundamental odd mode, and its resonance frequency is calculated by inserting the parameter values from Equation (21). Substituting the corresponding length values from Figure 7 into Equation (21) and referencing Figure 6c yields a theoretical frequency of 6.9 GHz for the second stopband, while the simulation shows 7.5 GHz. The slight deviations between the theoretical and simulated frequencies stem from the magnetic coupling between the resonators, which are adjusted for specific band applications through parametric analysis in the HFSS software.

Now, for the even mode, the input admittance can be calculated by making the stub short-circuit due to already being present; thus, *Y_L_* = α and *Y*_0_ = $\left(\frac{Y_{6}}{2}\right)$. Now, the equation above can be rewritten as

(17) $$Y_{in\text{-}shorted}=-j(\frac{Y_{6}}{2})\cot\theta_{6}$$

Putting Equation (17) in (15), Y_in-even_ can be obtained in the following equation:

(18) $$Y_{in,even}=-jY_{1}\frac{Y_{6}-2Y_{1}\tan(\theta_{5}+\theta_{7})\tan(\theta_{6})}{2Y_{1}\tan(\theta_{6})+Y_{6}\tan(\theta_{5}+\theta_{7})}$$

At resonance, *Y_in-even_* = 0, so the first stopband frequency (*f_even_*) equation is obtained using Figure 6a.

(19) $$f_{even}=\frac{(2n-1)c}{4(L_{5}+L_{6}+L_{7})\sqrt{\varepsilon_{eff}}}$$

In the above equation, *c* = 3 × 10^8^ m/s, and n is the integer, which is 1, 2, 3, …….. in this case, and $\varepsilon_{eff}$ is the PCB permittivity with the corresponding width (*w*) and height (*h*) which is

$$\varepsilon_{eff}=\frac{1+\varepsilon_{r}}{2}+\frac{\varepsilon_{r}-1}{2}\times \frac{1}{\sqrt{1+12\frac{h}{w}}}$$

Similarly, in the case of the odd mode, the stub *L*_1_ is short-circuited at AA′. The input admittance, *Y_in-odd_*, is obtained as follows by putting *Y*_0_ = *Y*_1_ and *Y_L_ = ∞* in Equation (4):

(20) $$Y_{in,odd}=\frac{Y_{1}}{j\tan(\theta_{5}+\theta_{7})}$$

The following equation of the second stopband is obtained by putting the resonance condition *Y_in-odd_* = 0 in the above equation.

(21) $$f_{odd}{}_{}=\frac{(2n-1)c}{4(L_{5}+L_{7})\sqrt{\varepsilon_{eff}}}$$

## 4. Proposed Filter Architecture 

A filter with stopband features with a circuit area of 1.12 λ_g_ × 0.6 λ_g_ has been designed and simulated in HFSS version 15. The structure is fabricated on a low-cost dielectric substrate, and a ZNB-20 vector network analyser (VNA) was used for testing the simulated results. The SUWB-BPF is made up of a UTL that has three-quarter-wavelength stubs loaded onto it; one of these stubs is placed on one upper side of the UTL, and the other is positioned symmetrically on the other side, while the filter with stopbands is designed using a shorted T-shaped resonator, coupled with the basic UWB structure. Figure 7 shows the layout of the proposed architecture with the dual-notch response, with dimensions in millimetres.

## 5. Hardware and Software Simulation Implementation

**Figure 8:**
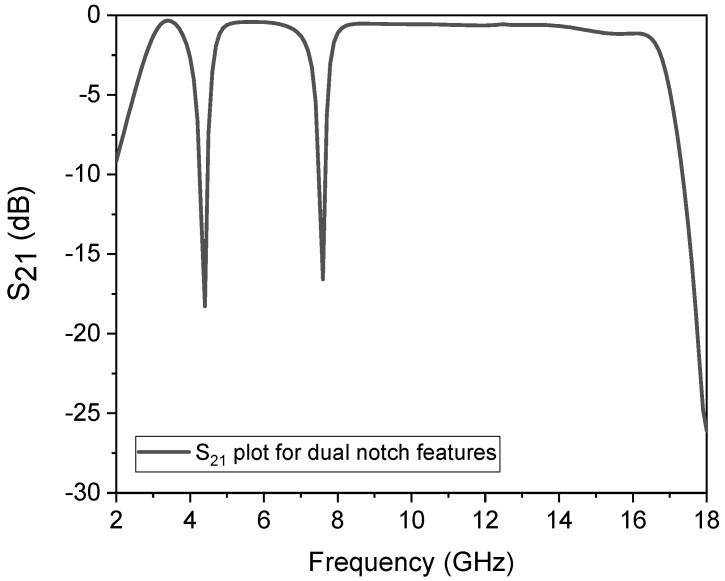
Simulated dual-stopband response.

In this study, the SWB BPF with stopband characteristics was designed and implemented. The SUWB-BPF is made up of a UTL that has three-quarter-wavelength stubs loaded onto it; one of these stubs is placed on one upper side of the UTL and the other is positioned symmetrically on the other side to achieve high sharpness in the filter and a flat passband response. A Rogers RO-4350 dielectric substrate with a low tangent loss of 0.0003, h = 1.5 mm, and *ϵ*_*r*_ = 3.6 was selected for the fabrication of the prototype to make the passband IL as low as possible, i.e., less than 0.4 dB, which leads to a flat group delay without ripples appearing in the passband. The structure was studied by considering a lossless transmission line and was investigated through the ABCD matrix. The replacement of conventional stubs with three new folded shunt stubs with pads enhances the filter’s performance in terms of its wide bandwidth, high selectivity, and multiple TPs. In the second step, two notches were created using a shorted, T-shaped resonator, coupled with the initial wideband filter, and analysed using a method called even–odd mode analysis. Two controllable stopbands were achieved for the application of long-distance wireless ISPs (WISPs), centred at 4.4 GHz, and X-band communication centred at 7.5 GHz. The first 4.4 GHz stopband was created due to the equivalent circuit shown in Figure 6b, while the second 7.5 GHz stopband was achieved due to the equivalent circuit shown in Figure 6c. The simulated response of the dual stopband filter is depicted in Figure 8.

**Figure 9:**
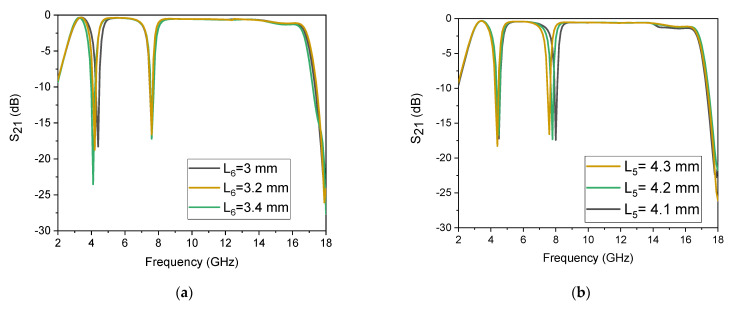
Notch band control: (**a**) response of the first stopband with controllable features; (**b**) response of the second stopband with controllable features.

It has the advantage that both stopbands can be controlled by the users, according to their application, based on the stubs’ length. For example, as shown in Figure 9a, the first stopband can be controlled using the central shorted stub with dimensions of *L*_6_ = 3 mm, and by changing the dimension, only the first stopband moves down, while the second band is stable. Similarly, from Figure 9b, the second stopband can be controlled by the outer half-wavelength stubs with dimensions of *L*_5_ = 4.2 mm, and by changing the dimension, only the second stopband changes while the first band is stable. So, this makes the proposed filter capable of stopping any frequency, according to the user’s applications.

**Figure 10:**
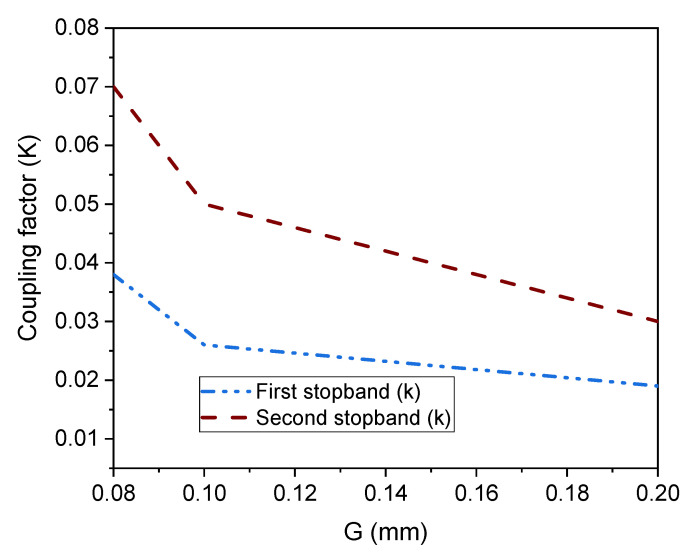
Coupling coefficient (k) plot with respect to the gap (G).

Another important phenomenon that needs to be discussed is the coupling coefficient (k) that appears when two or more resonators come in close contact [48]. As shown in Figure 7, the coupling phenomena originate based on the gap (G), which is given in Equation (22). As depicted in Figure 10, the “K” drops when the gap is increased from 0.08 mm to 0.2 mm, or vice versa.

(22) $$k\;=\frac{f_{2}{}^{2\;}-f_{1}{}^{2}}{f_{2}{}^{2\;}{+f}_{1}{}^{2}}$$

where f_1_ and f_2_ represent the lower and upper stopband frequency.

In the design of wideband filters or antennas, it is important to discuss the parameter group delay (*τ_d_*) and phase velocity (*φ*), which should be constant throughout the passband to avoid any frequency distortions, and can be expressed as [48]

(23) $$\Gamma_{d}=\frac{d\varphi_{21}(\omega )}{-d\omega }$$

(24) $$\varphi_{21}(\omega )=\arg S_{21}(\omega )$$

**Figure 11:**
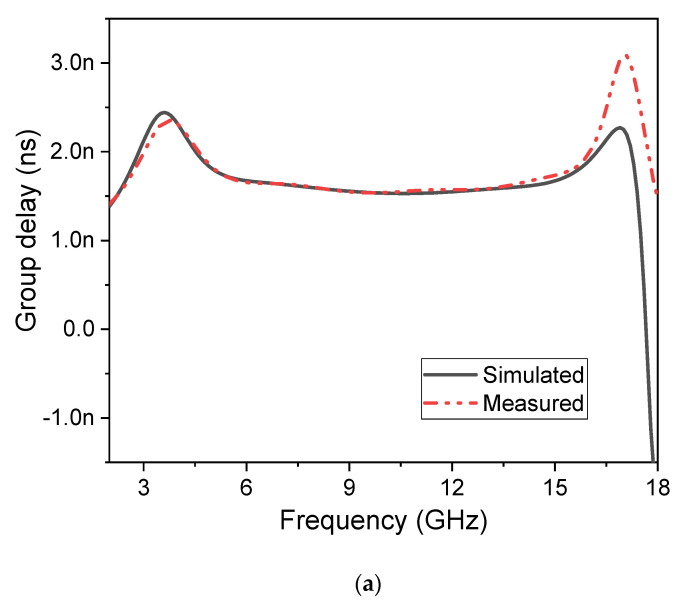

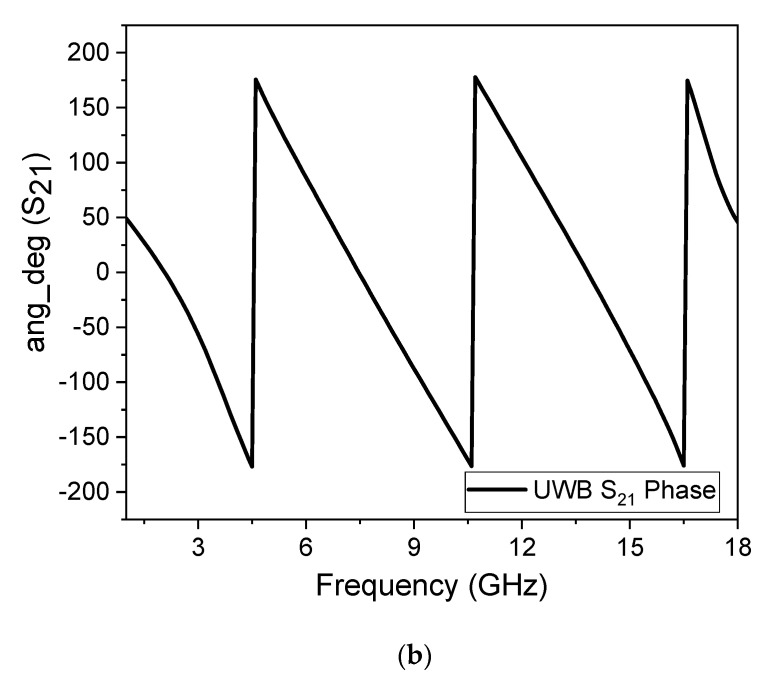
(**a**) Frequency vs. group delay response. (**b**) Frequency vs. phase response of the SWB filter.

The terms *φ*_21_ and *τ_d_* in the equations above stand for the parameter phase and group delay, relative to the magnitude (*S*_21_). As shown in Figure 11a, the group delay is practically flat across the full passband, ensuring that all current flows with the same velocity and phase, resulting in the minimum amount of frequency dispersion possible. Also shown in Figure 11b is the suggested UWB filter’s phase response.

**Figure 12:**
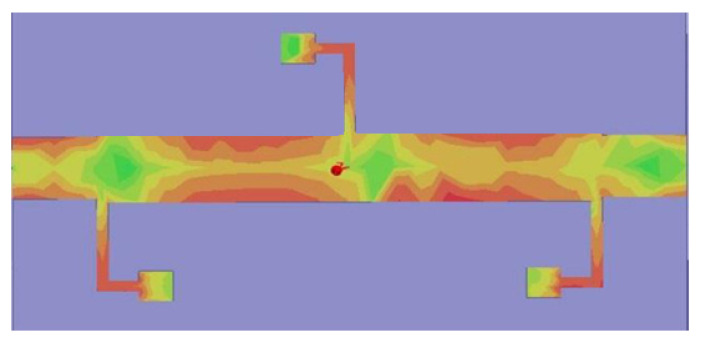
Current distribution graph of the ultra-passband at 9.65 GHz.

The distribution of currents in the passband, which is utilized to confirm the resonant behaviour of the ultra-wideband filter, is another important hypothesis that should be explained. The simulated current distributions at a centre frequency of 9.65 GHz are shown in Figure 12. As discussed, the wideband filter is constructed on the UTL with three folded λ/4 shunt stubs, as shown in Figure 1; therefore, it is expected that most of the current energy will be absorbed by this portion of the filter. This absorption of current energy by the UTL and stubs contributes to the creation of the passband in the filter.

**Figure 13:**
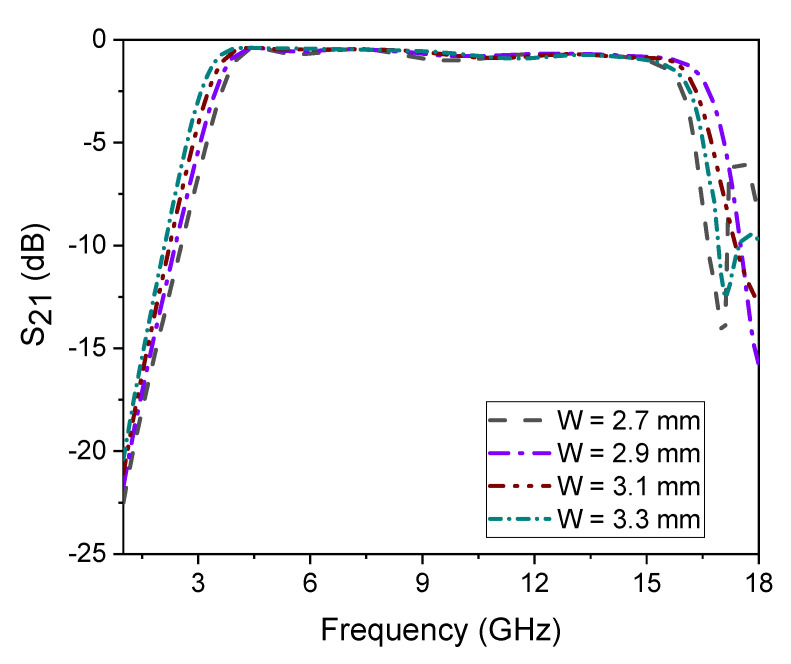
Bandwidth control with different values of parameter W.

**Figure 14:**
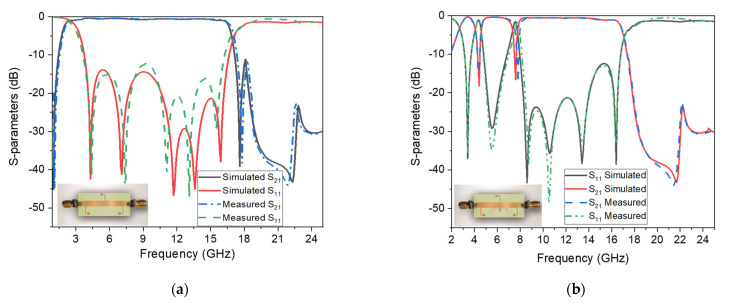
Simulated and measured S11 and S21 frequency plots. (**a**) The proposed SWB BPF. (**b**) The proposed SWB BPF with dual stopbands.

The simple architecture to decrease costs and the configurable bandwidth for user end selection are stated to be innovative in this work. As seen in Figure 13, the wideband filter’s bandwidth changes by varying the uniform transmission line’s width. To evaluate the performance of the proposed structure, a comparison is made with the recently published filters in the literature. The comparison is based on various parameters tabulated in Table 1. By presenting this comparison, the authors highlight the advantages of their proposed structure and demonstrate its competitiveness in terms of key performance metrics. This provides further support for the novelty and effectiveness of the research work. Moreover, the S-parameter plots with photographs of the proposed SWB filter and the SWB filter with dual-notched bands are shown in Figure 14a,b, respectively. The small discrepancy between the experimental and manufactured findings is caused by measurement errors made by humans that are unavoidable, as well as the effect of soldering, losses in the substrate material, and the SMA connectors.

## 6. Conclusions

In this study, a SWB BPF with dual stopband features at 4.4 GHz and 7.5 GHz has been designed using a UTL loaded with three-λ/4 stubs and a coupled T-shaped resonator. The operating principle of such a filter was explained in detail, and its controllable broadband response with notch band features was successfully achieved to show some attractive features, such as a wide bandwidth of about 14.3 GHz, a simple topology, a flat group delay, a low insertion loss of 0.4 dB, a high return loss of greater than 10 dB, and sharp rejection at the lower and upper cut-off frequencies, with good out-of-band performance up to 25 GHz, respectively. These characteristics enable the integration of the proposed filter into upcoming wireless communication systems.

## Figures and Tables

**Table 1:** Comparisons with other reported work.

Ref. No.	Passband (GHz)	FBW (%)	IL/RL (dB)	C.F/BW (GHz)
[11]	1.8–3.1	62.5	0.5/20	2.45/1.3
[12]	2.7–8.23	132%	2.5/10	5.46/5.53
[14]	2.92–10.95	107	0.49/>12	6.93/8.03
[49]	3.21–10.77	109.4	0.8/15	8.6/7
[50]	1.44–2.66	60	0.6/20	2.05/1.22
[51]	1.64–2.47	40	0.8/20	2.05/0.83
[52]	2.3–4.08	50.3	1.2/12	6.38/1.78
[53]	2.4–7.2	83	0.5/14	6/4.8
[54]	2.94–10.39	111.6	0.5	6.66/7.45
[55]	3.1–10.6	109	<0.5	6.85/7.5
[56]	3.1–10.6	119	0.35	6.85/7.5
[57]	3.7–9.6	106.2	<1	6.65/3.2
[58]	3.6–10.4	103.9	>0.5	7/6.8
[59]	2.2–2.53	4	2.5/>20	2.3/0.33
[60]	1–3	135	0.1/>20	2/2
[61]	2.95–10.75	113.9	0.6/14	6.85/7.8
[62]	3.05–10.62	100.9	1.5/13	6.83/7.57
[63]	9.5–10.5	8.5	1.8/>10	10/1
**This work**	**2.5–16.8**	**148.18**	**>0.4/>10**	**9.65/13.9**

## Data Availability

Not applicable.
